# Investigating intergroup attitudes in Europe: Cross-national data on news media, attitudes towards newcomers, and socio-psychological indicators

**DOI:** 10.1016/j.dib.2019.104535

**Published:** 2019-09-17

**Authors:** David De Coninck, Leen d’Haenens, Willem Joris

**Affiliations:** a Centre for Sociological Research, KU Leuven, Leuven, Belgium; b Institute for Media Studies, KU Leuven, Leuven, Belgium

**Keywords:** Attitudes towards newcomers, News media consumption, News media trust, Europe, Refugees

## Abstract

This dataset is part of a multidisciplinary project that investigates the relationship between attitudes of the adult population towards ‘newcomers’, and news media consumption and trust in Belgium, France, the Netherlands, and Sweden in 2017 (N = 6000). These countries were chosen for their response to the 2015 refugee crisis in Europe, with each country having a different media context. We ran an online survey which included questions on socio-demographic characteristics, attitudinal indicators, and information on news media consumption and trust. The data are representative for age and gender in each country and can be of interest for researchers who wish to explore dynamics of attitude formation, particularly with regards to the role of news media, in the wake of the 2015 refugee crisis in Europe.

**Table:** Specifications Table

Subject	Sociology and Political Science; Media Studies
Specific subject area	Attitudes towards immigrants and refugees; News media consumption and trust
Type of data	Table
How data were acquired	Online survey among the adult population in Belgium, France, the Netherlands, and Sweden.
Data format	Raw, analysed and filtered.
Parameters for data collection	Being over the age of 17 and under the age of 66 and residing in Belgium, France, the Netherlands, or Sweden at the time of the fieldwork (September through October of 2017).
Description of data collection	We collected the data in cooperation with a Belgian polling agency and selected the methodology for its cost-effectiveness in cross-country research. This is also related to the country selection: the polling agency we worked with has a strong presence in the four countries, which facilitated fieldwork efforts. Respondents received an e-mail asking them to participate in a survey without specifying the subject matter, which was essential to avoid priming. Three weeks of fieldwork in September and October of 2017 resulted in a dataset of 6000 respondents (1500 per country). Sample weights were applied to ensure representativeness of the sample for gender and age in each country. The response rate was about 35%.
Data source location	Institution: KU Leuven City/Town/Region: Leuven Country: Belgium
Data accessibility	Repository name: Investigating intergroup attitudes in Europe Data identification number: https://doi.org/10.17632/3m439mbrmm.2 Direct URL to data: https://data.mendeley.com/datasets/3m439mbrmm/2

**Table:** 

**Value of the Data** • The data presented can help researchers to better understand the relationship between individual attitudes towards newcomers (immigrants and refugees), and the consumption of and trust in specific news media channels in Belgium, France, the Netherlands, and Sweden in the wake of the European refugee crisis. • Researchers of intergroup attitudes can benefit from these data because they highlight how attitudes differ between specific groups of newcomers (immigrants vs. refugees), and they can also provide a comparative perspective with measurements that were carried out prior to the refugee crisis. • Researchers of media studies can benefit from the detailed measurements of news media consumption and trust, as differences between public and commercial broadcasters and quality and popular newspapers are highlighted. • These data can be used to further develop a framework on intergroup attitudes with a stronger emphasis on media, an aspect which is often insufficiently considered to influence attitudes in sociological research. • The data presented are rare: there is very little research that combines indicators on intergroup attitudes with measures on news media. This allows for new insights in a field which is rapidly evolving, and where researchers and policy makers alike are looking for new insights.

## 1 Data

The data presented in this article were collected in the context of two projects: Image of Immigrants in the Media: Thought-Provoking Effects (IM^2^MEDIATE) and Focusing on Refugees and Inclusion in European News Diversity Studies (FRIENDS). The aim of these projects is to investigate the dynamic interplay between media representations of the current non-EU immigrant situation with a specific emphasis on the refugee situation on the one hand and the governmental and societal (re)actions on the other. With these data, we provide more insight into these societal reactions by investigating attitude formation. Through an online survey, we collected quantitative data on attitudes towards newcomers, news media consumption, frequency and valence of direct intergroup contact, threat frames, and socio-demographic characteristics for the adult population aged 18 to 65 in four European countries: Belgium, France, the Netherlands, and Sweden. We collected the data in cooperation with a Belgian polling agency and selected the methodology for its cost-effectiveness in cross-country research. This is also related to the country selection: the polling agency we worked with has a strong presence in the four countries, which facilitated fieldwork efforts. Adding additional countries was desirable, but the financial means for this were not available at the time. Respondents received an e-mail asking them to participate in a survey without specifying the subject matter, which was essential to avoid priming. Three weeks of fieldwork in September and October of 2017 resulted in a dataset of 6000 respondents (1500 per country). Sample weights were applied to ensure that the sample is representative for gender and age in each country. The response rate was about 35% [1,2]. Table 1 shows the distribution of respondents by several socio-demographic characteristics, while Table 2 presents mean scores of selected indicators on attitudes, news media consumption and trust, threat, and intergroup contact.

**Table 1:** Socio-demographic characteristics of participants in the four countries (in %, unless otherwise specified).

	Belgium N = 1500	France N = 1500	Netherlands N = 1500	Sweden N = 1500	Total N = 6000
**Gender**					
Male	50.3	49.0	49.0	50.0	49.6
Female	49.7	51.0	51.0	50.0	50.4
Household income*					
Low income (Under €2500)	44.5	57.0	57.2	33.1	48.1
Average income (€2500 – €4500)	44.5	32.7	34.6	35.8	36.7
High income (Over €4500)	11.0	10.4	8.2	31.2	15.2
**Mean age** (in years)	42.9	43.5	45.6	41.4	43.4
**Educational attainment**					
No degree/Primary degree	4.1	3.1	4.2	8.0	4.8
Secondary degree	42.8	52.4	57.0	54.2	51.6
Tertiary degree	53.1	44.5	38.7	37.9	43.5
**Religious denomination**					
Christian	49.9	51.5	42.8	32.2	44.2
Muslim	0.7	3.9	2.1	4.6	2.8
Other	2.1	1.2	2.8	2.0	2.1
Freethinking	9.0	6.3	1.1	5.9	5.6
Not religious	38.3	37.0	51.2	55.3	45.4
**Religious piety**					
Non-believer/not active	61.7	51.7	61.5	46.3	55.2
Believer	34.7	41.5	25.9	46.1	37.0
Religious	3.3	4.9	9.3	5.5	5.8
Pious	0.4	1.9	3.3	2.2	2.0

**Table 2:** Mean scores of selected indicators on attitudes, news media consumption and trust, threat, and intergroup contact.

	Belgium N = 1500	France N = 1500	Netherlands N = 1500	Sweden N = 1500	Total N = 6000
**Attitudes towards immigrants**					
Same ethnicity	2.82	2.53	2.71	2.97	2.76
Different ethnicity	2.47	2.29	2.50	2.65	2.48
From rich European countries	2.71	2.49	2.57	2.83	2.65
From poor European countries	2.56	2.36	2.45	2.73	2.52
From rich non-European countries	2.49	2.32	2.46	2.70	2.50
From poor non-European countries	2.38	2.25	2.37	2.63	2.41
From Muslim countries	2.31	2.19	2.23	2.53	2.32
**Attitudes towards refugees**					
Same ethnicity	2.79	2.53	2.69	2.94	2.74
Different ethnicity	2.52	2.34	2.55	2.70	2.53
From rich European countries	2.60	2.44	2.52	2.78	2.58
From poor European countries	2.57	2.38	2.53	2.74	2.56
From rich non-European countries	2.47	2.35	2.47	2.68	2.49
From poor non-European countries	2.47	2.30	2.50	2.67	2.48
From Muslim countries	2.36	2.21	2.31	2.65	2.36
**News media consumption**					
Public television	4.28	2.90	4.94	4.27	4.10
Commercial television	3.78	3.64	4.42	4.17	4.00
Public radio	4.02	1.98	2.56	3.23	2.95
Commercial radio	3.10	2.75	3.53	3.20	3.14
Quality newspaper*	1.51	1.39	1.37	1.81	1.52
Popular newspaper*	1.72	1.66	1.95	2.80	2.03
Quality online news*	2.10	1.55	1.50	2.05	1.80
Popular online news*	2.36	1.48	2.59	3.67	2.52
Social media*					
**Trust in news media**					
Public television	3.79	3.03	3.58	3.31	3.43
Commercial television	3.32	2.93	3.26	3.10	3.15
Public radio	3.73	3.04	3.48	3.31	3.39
Commercial radio	3.27	3.03	3.22	2.62	3.03
Quality newspaper*	3.65	3.05	3.44	3.20	3.34
Popular newspaper*	3.23	2.96	3.14	2.62	2.99
Online news media	3.40	2.98	3.26	2.86	3.12
Social media	2.44	2.50	2.64	2.27	2.46
**Realistic threat**					
Jobs	5.33	5.15	5.43	6.49	5.60
Social Security	4.77	4.83	4.90	5.18	4.92
Economy	5.02	4.73	5.36	5.45	5.14
Wages	2.98	3.42	2.75	2.66	2.95
**Cultural threat**	5.57	5.03	5.78	6.26	5.66
**Fear of terrorism**					
I may be on vehicle that is bombed	5.81	7.91	4.49	6.14	6.09
I may be victim of terror attack	6.05	8.02	4.82	6.41	6.32
I may witness terror attack	6.23	8.10	5.07	6.58	6.49
Family is on vehicle that is bombed	6.34	8.16	5.07	6.54	6.53
Family is victim of terror attack	6.43	8.16	5.16	6.59	6.58
Family witnesses terror attack	6.64	8.19	5.34	6.72	6.72
**Intergroup contact**					
Friends	2.18	2.00	2.19	1.91	2.07
Random	4.97	4.42	4.47	5.36	4.81
**Valence intergroup contact**	7.37	7.26	7.71	7.15	7.37

Note. Variables denoted with * indicate the average consumption based on the scores of several individual newspapers/websites.

## 2 Experimental design, materials, and methods

Although several public survey platforms exist that measure public opinion on newcomers, insurmountable shortcomings in these national and international surveys stimulate us to collect new data, which includes information on news media exposure and news media trust (among others) alongside variables measuring public opinion/attitudes towards immigrants and refugees. To recapitulate, we developed an online public opinion survey, to be carried out amongst a sample of Belgian, French, Dutch and Swedish residents aged 18 to 65, representative for gender and age. The survey was distributed by iVOX, a market research and online polling agency active in all countries included in the dataset. The fieldwork included the use of incentives to maximize the response rate. Participants who completed the survey were entered into a pool to win a tablet at the end of the fieldwork period. The survey we have developed consists of eight themes: socio-demographic characteristics, media- and news consumption, trust in media, attitudes towards immigrants, contact with and knowledge on immigrants, attitudes towards and knowledge on refugees, culture of fear and psychological indicators. In what follows, we highlight several measures. All data were processed and cleaned through SPSS.

### 2.1 Attitudes towards immigrants and refugees

As to accurately measure the difference in public opinion on immigrants and refugees, we decided to adapt a scale previously used in a rotating module of the European Social Survey. This module was created to measure immigration attitudes and was included in Round 1 (2002) and Round 7 (2014). The adapted scale consists of seven items asking which groups of immigrants should be allowed to come and live in Belgium: ‘*Immigrants of the same race or ethnicity as most of the country's population*.’; ‘*Immigrants of a different race or ethnicity as most of Belgium's population*.’; ‘*Immigrants of the richer countries in Europe.”*; *“Immigrants of the poorer countries in Europe*.’; ‘*Immigrants of the richer countries outside Europe.*’; ‘*Immigrants of the poorer countries outside Europe.*’; and *‘Immigrants coming from Muslim countries who wish to work in Belgium.*’ Answer categories range from 1 (Allow none) to 4 (Allow many). To collect the data, we presented the scale in its original form and added an extra item concerning immigrants from Muslim countries. The reason for the inclusion of this item lies in the fact that a majority of immigrants and refugees entering Europe in the current refugee crisis originate from Syria, Iraq or Afghanistan – predominantly Muslim countries [3]. Prior to completing each block of items, respondents were presented with a definition of immigrants and refugees from the United Nations with the request they keep this definition in mind during completion of the survey.

The definition of immigrants was as follows:

An immigrant should be understood as covering all cases where the decision to migrate is taken freely by the individual concerned, for reasons of ‘personal convenience’ and without intervention of an external compelling reason (e.g., war, natural disaster) [4]. (UNESCO, 2017, para. 3)

The definition of refugees was:

A refugee is someone who has been forced to flee his or her country because of persecution, war, or violence. A refugee has a well-founded fear of persecution for reasons of race, religion, nationality, political opinion or membership in a particular social group [5]. (United Nations, 1951, p. 14)

We clearly highlighted these two definitions so that respondents would be able to distinguish between immigrants and refugees and provide a reliable measurement of attitudes for each group [5,6]. As evidenced by Table 2, mean scores indicate there are clear differences across the countries, French residents being most negative and Swedish residents being most positive towards both groups of newcomers. There are also differences between groups of newcomers: respondents hold more positive attitudes towards refugees than towards immigrants and are more likely to accept newcomers from the same ethnicity or originating from rich (European) countries.

### 2.2 News media consumption and trust

Respondents were asked about their media consumption patterns during the past month, with answer categories ranging from 0 (Never) to 7 (Every day). Both television and radio consumption were split into two groups: public and commercial broadcasters. For the classic newspaper and online news consumption, the most commonly read newspapers and commonly visited webpages in each region were included separately. This selection of newspapers was based on information concerning the circulation of newspapers in each country. Online and social media were also included. For online media, most of the websites of the newspapers that were included were selected, with some additional online-only news outlets. As for social media, the most commonly used media in 2017 were included.

We measured trust in the news media brands by means of a five-point scale with answer categories ranging from 1 (No trust at all) to 5 (A lot of trust). Like the methodology for news media consumption, trust was measured separately for public and commercial television and radio. Trust in newspapers was aggregated into two groups: trust in quality newspapers and trust in popular newspapers to avoid multicollinearity in the data. Trust in news websites/apps, and trust in social media were also measured on the same scale.

### 2.3 Threat perspectives and intergroup contact

In the measurement on threat perspectives, we included several types of threat. A first set of indicators assesses economic (or realistic) threat. This was measured by asking four questions: ‘*Would you say that refugees who come to live here generally take jobs away from workers in [country], or generally help to create new jobs?*’, ‘*Most refugees who come to live here work and pay taxes. They also use health and welfare services. On balance, do you think refugees who come here take out more than they put in or put in more than they take out?*’, and *‘Would you say it is generally bad or good for [country]'s economy that refugees come to live here from other countries?*’. One additional item on realistic threat is ‘*Average wages and salaries are generally brought down by refugees coming to live and work here.’* Another threat perspective is on cultural threat and was measured through the following question: ‘*Would you say that [country]'s cultural life is generally undermined or enriched by refugees coming to live here from other countries?*’. Both types of threat were measured on an 11-point scale, with the high end of the scale signifying a low degree of threat. The only exception is the item on wages and salaries being brought down by refugees coming to live in the country, as this is coded on a five-point scale with the high end of the scale indicating strong agreement with this statement. A third type of threat is on fear of terrorism, which was particularly relevant to include given the current events in Europe around the time of the survey. We adapted the fear of terrorism-scale developed by Nellis and Savage [6]. The scale consists of several hypothetical scenarios (‘*Someday I may witness a terror attack’*, *‘Someday I may be the victim of terror attack*’, …), with answer categories ranging from 0 (Not likely at all) to 10 (Very likely). Whereas the original scale measured this fear for several scenarios separately *(‘I could be on a plane that is hijacked*’, ‘*I could be on a subway or bus that is hijacked’*), we have aggregated these into a single item (‘*Someday I may be on a plane/subway/bus that is bombed’*). Some of the original wording was also adjusted.

Direct intergroup contact was measured by asking whether respondents have any interethnic friendships (no, some, or many), and how often they have interethnic random contact on the street, at work, in shops… [seven categories, ranging from 0 (Never) to 6 (Every day)]. The valence of direct contact was measured by an 11-point scale, with 0 indicating a negative evaluation of intergroup contact, and 10 indicating a positive evaluation. These indicators on economic and cultural threat, and on direct intergroup contact stem from the rotating module on immigration in Round 1 and Round 7 of the European Social Survey [7].
